# Supplementary material for: The Systems Biology Research Tool: evolvable open-source software
Source: BMC Syst Biol. 2008 Jun 29;2:55. doi: 10.1186/1752-0509-2-55 (PMC2446383; doi:10.1186/1752-0509-2-55)
Supplement: Additional file 1 — SBRT Archive. An archive of the current version of the Systems Biology Research Tool. [file 1752-0509-2-55-S1.zip › sbrt-1.4.0/doc/developers_guide/api/sbrt/kernel/fba/class-use/CatalyzedFluxome.html]

Uses of Interface sbrt.kernel.fba.CatalyzedFluxome


|  |  |  |
| --- | --- | --- |
| **Overview** \| **Package** \| **Class** \| **Use** \| **Tree** \| **Deprecated** \| **Index** \| **Help** | |  |
| PREV   NEXT | **FRAMES**    **NO FRAMES**     **All Classes** |  |


---


## **Uses of Interface sbrt.kernel.fba.CatalyzedFluxome**

| Packages that use CatalyzedFluxome | |
| --- | --- |
| **sbrt.kernel.fba** | Provides classes and interfaces for creating objects relevant to Flux Balance Analysis. |
| **sbrt.kernel.fba.proc** | Provides classes and interfaces for executing Flux Balance Analysis processes. |
| **sbrt.shell.mng.fba** | Provides classes and interfaces for creating Flux Balance Analysis process managers. |
| **sbrt.shell.text.fba** | Provides classes and interfaces for defining formats for objects relevant to Flux Balance Analysis. |

Uses of CatalyzedFluxome in sbrt.kernel.fba

| Subinterfaces of CatalyzedFluxome in sbrt.kernel.fba | |
| --- | --- |
| `interface` | `IrrevRxnFluxome`             This class is used to represent fluxomes that contain only irreversible reactions. |

| Classes in sbrt.kernel.fba that implement CatalyzedFluxome | |
| --- | --- |
| `class` | `IrrevRxnFluxomeV1`             This class is a concrete implementation of IrrevRxnFluxome. |

| Methods in sbrt.kernel.fba that return CatalyzedFluxome | |
| --- | --- |
| `CatalyzedFluxome` | `CatalyzedFluxome.replaceConstraints(FluxConstraints constraints, double constraintTolerance)`             Returns a copy of this fluxome, but with all constraints replaced by those provided. |

Uses of CatalyzedFluxome in sbrt.kernel.fba.proc

| Methods in sbrt.kernel.fba.proc that return CatalyzedFluxome | |
| --- | --- |
| `CatalyzedFluxome` | `NetworkInfoProcess.getFluxome()`             Returns the fluxome for which information is to be gathered. |

Constructors in sbrt.kernel.fba.proc with parameters of type CatalyzedFluxome

`NetworkInfoProcess(CatalyzedFluxome fluxome)`
  
          Constructs a new flux activity experiment.

Uses of CatalyzedFluxome in sbrt.shell.mng.fba

| Methods in sbrt.shell.mng.fba with parameters of type CatalyzedFluxome | |
| --- | --- |
| `static InputFile<java.util.Set<java.lang.String>>` | `CatalystDeletionManager.getDeletionFile(java.lang.String fileName, CatalyzedFluxome fluxome)`             Returns the catalyst input file with the provided name. |
| `static InputFile<java.util.Set<java.lang.String>>` | `CatalystDeletionManager.getDeletiontFile(ManagerInput<java.lang.String,java.lang.String> input, java.io.PrintWriter out, CatalyzedFluxome fluxome)`             Parses and returns the value for `CatalystDeletionManager.CATALYST_DELETION_FILE` in the provided manager input. |

Uses of CatalyzedFluxome in sbrt.shell.text.fba

| Methods in sbrt.shell.text.fba that return CatalyzedFluxome | |
| --- | --- |
| `CatalyzedFluxome` | `CatalystVerifier.getFluxome()`             Returns the fluxome used to create this catalyst name verifier. |
| `CatalyzedFluxome` | `CatalystListFormatV1.getFluxome()`             Returns the fluxome used to verify catalyst names. |
| `CatalyzedFluxome` | `CatalystSetFormatV1.getFluxome()`             Returns the fluxome used to verify catalyst names. |
| `CatalyzedFluxome` | `CatalystListFormat.getFluxome()`             Returns the fluxome used to verify catalyst names. |

Constructors in sbrt.shell.text.fba with parameters of type CatalyzedFluxome

`CatalystListFormatV1(CatalyzedFluxome fluxome)`
  
          Constructs a new catalyst name collection format
using the provided fluxome.

`CatalystSetFormatV1(CatalyzedFluxome fluxome)`
  
          Constructs a new catalyst name collection format
using the provided fluxome.

`CatalystVerifier(CatalyzedFluxome fluxome)`
  
          Constructs a new catalyst name verifier for the provided fluxome.

---


|  |  |  |
| --- | --- | --- |
| **Overview** \| **Package** \| **Class** \| **Use** \| **Tree** \| **Deprecated** \| **Index** \| **Help** | |  |
| PREV   NEXT | **FRAMES**    **NO FRAMES**     **All Classes** |  |


---
